# More Than Just an Ulcer: A Case of HIV-Associated Iliac Aneurysms Presenting With a Leg Ulcer

**DOI:** 10.7759/cureus.13203

**Published:** 2021-02-07

**Authors:** Sherif Roman, Nader Mekheal, Shani Tal, Patrick Michael

**Affiliations:** 1 Internal Medicine, St. Joseph's Regional Medical Center, Paterson, USA; 2 Internal Medicine, St. Joseph's Univeristy Medical Center, Paterson, USA

**Keywords:** human immunodeficiency virus, hiv-vasculopathy, atherosclerosis, hiv-aneurysms, occlusive disease, iliac aneurysm, incidental aneurysm, haart, leukocytoclastic vasculitis

## Abstract

The incidence of symptomatic vasculitis in human immunodeficiency virus (HIV)-infected patients is approximately 1%, and it commonly presents as arterial occlusive disease or aneurysmal disease. Early diagnosis of vascular complications in those patients is essential; however, it is extremely challenging. Iliac aneurysms are usually silent, and because of their deep location, detection of these aneurysms is typically difficult. Therefore, they always continue to be asymptomatic until rupture unless they are discovered incidentally on a radiological investigation for an irrelative condition. We present the case of a 61-year-old HIV-positive man with bilateral iliac aneurysms and total coronary artery occlusion presenting with a leg ulcer.

## Introduction

Human immunodeficiency virus (HIV) infection remains a significant health burden, affecting approximately 40 million people worldwide [1]. It is involved in a multisystem disease process, including the cardiovascular system. Many patients present with vascular complications in the late stages of the disease [2].

Human immunodeficiency virus-associated vasculopathy was first defined in 1987 [3]. It can present with a broad spectrum of occlusive disease, aneurysms, dissections, and spontaneous arteriovenous fistulae. After introducing HAART to the clinical practice, overall survival appears to be markedly improved; however, this improvement is negated by the possible vascular complications.

As a result, the incidence of vasculopathic complications such as arterial occlusive disease and aneurysmal disease has increased [4]. With pathogenesis that is still not well understood, HIV is associated with cardiovascular complications, including coronary artery disease (CAD), myocarditis, and arterial aneurysms. The clinical presentation of these aneurysms, particularly iliac aneurysms, can be challenging and requires high clinical suspicion. In this report, we present the case of a 61-year-old HIV-positive man with bilateral iliac aneurysms and total coronary artery occlusion presenting with a leg ulcer. The patient has no past medical history of diabetes or cardiovascular diseases.

## Case presentation

A 61-year-old African male from Kenya presented to our facility complaining of a worsening nonhealing leg ulcer associated with pain, intermittent fever, and purulent discharge. He attributed his wound to having a low-impact trauma two weeks ago, and it continued to worsen despite antibiotic treatment.

He reported hypoesthesia and mild intermittent claudication symptoms for the past several months. The patient tested HIV-positive in 2008 and endorsed compliance with his antiretroviral therapy. He is an active smoker with no history of diabetes or cardiovascular diseases. The patient also has bilateral venous stasis dermatitis.

On examination, the patient was afebrile. A 4 cm x 5 cm shallow ulcer was noted superior to the left lateral malleolus draining purulent discharge, with another small ulcer on the left medial malleolus in addition to lipodermatosclerosis bilaterally. Laboratory analysis showed a normal white cell count of 6.1 × 103 µL with elevated erythrocyte sedimentation rate 92 mm/h, C-reactive protein 58.2 mg/L, CD4 lymphocyte count 730/mm3, and HIV RNA level 40 copies/mL and wound culture grew *Escherichia coli*.

Investigations

**Figure 1:**
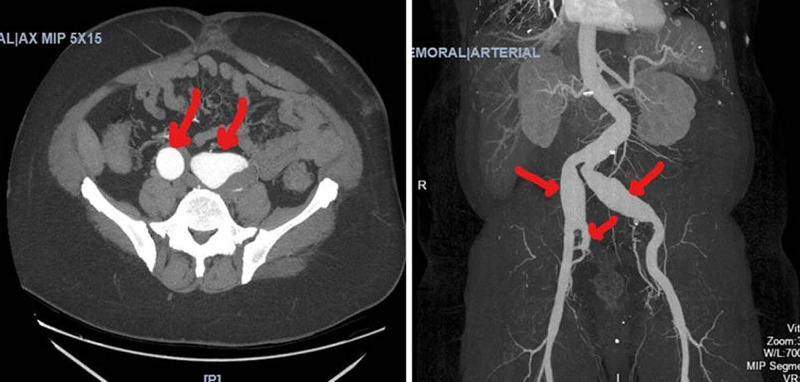
CT with contrast showing bilateral common iliac artery aneurysms and right internal iliac aneurysm.

Initially, venous ultrasound was performed, which revealed a nonocclusive echogenic thrombus in the right popliteal vein. Then, a CT venogram was done and showed tortuous abdominal aorta and bilateral common iliac artery aneurysms. A right common iliac artery aneurysm measures 3.5 cm x 3.3 cm and consists of mostly flowing blood lumen, the left common iliac artery aneurysm contains more thrombus and measures 4.8 cm x 5.3 cm and another 2.5 cm aneurysm of the right internal iliac artery. A CT angiography subsequently confirmed the CT venogram findings (Figure 1).

Endovascular repair was planned; however, preoperative stress test showed a significant defect involving the inferoseptal segment and septal area. Subsequently, cardiac catheterization was performed and demonstrated single-vessel CAD with chronic total occlusion of the proximal segment of left anterior descending artery (LAD) with good collateral circulation; nonobstructive disease.

Treatment and outcome

**Figure 2:**
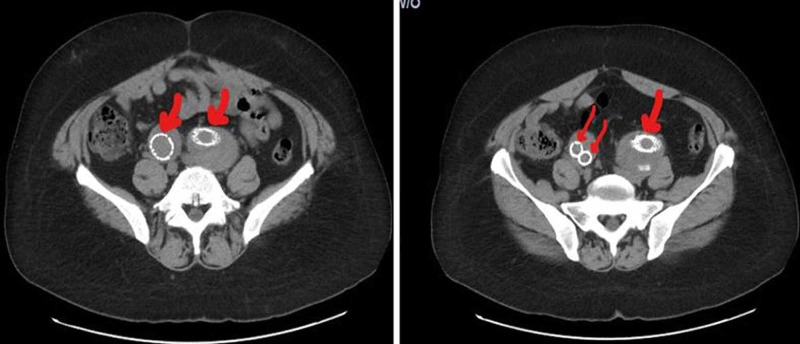
CT with contrast showing bilateral common iliac artery aneurysms and right internal iliac aneurysm after endovascular repair.

The patient was started on medical therapy for CAD; meanwhile, his leg ulcer had improved after IV antibiotics. The patient was discharged from the hospital, and three weeks later, he underwent endovascular repair of bilateral common iliac artery aneurysms and coil embolization of the right internal iliac artery (Figure 2).

He had a stable postoperative course with marked improvement in symptoms, including leg pain, intermittent claudication, and tissue healing.

## Discussion

The HIV poses a significant health concern by affecting approximately 1.2 million people in the United States [5]. Patients tend to have a wide range of complications regarding all organs of the body. With pathogenesis still not well understood, some cardiovascular complications include CAD, myocarditis, arterial aneurysms, vasculitis, and many other conditions are associated with patients diagnosed with HIV.

The early detection of those vascular complications is quite challenging. Our patient presented with a leg ulcer after a low-impact trauma; however, the iliac aneurysms and the coronary artery occlusion were incidentally found during the investigations. One of the possibilities of venous leg ulcer presentation can be due to compression of the iliac arteries on the veins leading to venous stasis and venous skin ulcers.

Both inflammatory and noninflammatory vasculitis have been associated with HIV patients, and they can be aneurysmal or occlusive, with the occlusive disease being less common. The aneurysms found in these patients were either single or multiple, and they can affect all major arterial vessels such as the aorta, common carotid, common iliac, femoral, or popliteal arteries.

The mechanism of aneurysm formation is still undetermined, but there are few theories with histopathologic evidence about how these aneurysms develop. In young patients with HIV, aneurysms formation is usually attributed to leukocytoclastic vasculitis of the vasa vasorum and periadventitial vessels without atherosclerosis. Additional theories include viral inflammation and mycotic destruction leading to weakening of the endothelial wall and forming aneurysms. Histology reveals inflammatory cell infiltrates with majority neutrophils, elastin fragmentation, and smooth muscle loss [4]. These patients were found to have small and narrow adventitial vessels with T cells detected in the adventitia, features that overlap with Takayasu arteritis, a subtype of large vessel vasculitis [6].

In another study performed by Nair et al., 60 aneurysms found in HIV patients were explored in detail to determine their etiology. Only 34% of them occurred with atherosclerotic features, while 64% of them had similar features to Takayasu arteritis, further indicating the similarity seen with this patient population and medium-sized vessel vasculitis [6].

Our patient also reported that he was compliant with his HAART medications, which could also increase the risk of developing his vascular complications. Since the use of HAART medications, the prevalence of cardiovascular complications has increased, which could be due to increased patients' survival with the disease. Additionally, many new antiretroviral agents, such as the protease inhibitors, tend to have metabolic side effects such as lipodystrophy, hyperglycemia, and dyslipidemia that would accelerate the risk of cardiovascular complications [7].

In a retrospective study that included about 5000 patients, there was a four-fold increase in the incidence of myocardial infarction among HIV-infected patients on HAART compared with the incidence among patients who underwent treatment before using HAART [8]. Another study reviewed both clinical and autopsy results and showed that the prevalence of myocardial abnormalities in HIV-positive patients ranged from 25% to 75% [9]. Of those treated with the classic antiretroviral regimen, there was a 26% increase in patients who suffered from a myocardial infarction, likely due to the medication's side effects and accelerated atherosclerosis [10].

Isolated aneurysms on the common iliac artery are rare, with an incidence of 0.008%-0.03% in the general population [11]. These patients can be asymptomatic, or they can experience compressive symptoms as the aneurysm enlarges with age. The most feared complication of an isolated common iliac aneurysm is rupture, which was found to occur in 31% of patients with aneurysms with a diameter of 5.6 cm [12]. Aneurysms measured to be larger than 3 cm are often scheduled for endovascular repair, which has a 90% survival rate [13]. In patients with iliac artery aneurysm detected accidentally, timely management is encouraged to avoid the complicated sequelae following rupture [14].

## Conclusions

Despite advances in HIV management, cardiovascular complications such as vessel occlusive disease and aneurysm formation have been increasing. Most of these aneurysms remain asymptomatic, and early detection of them is essential but deemed very challenging. Physicians should be aware of the disease process and have high clinical suspicion to promptly diagnose and manage these complications. Further research is still needed to better understand the pathophysiology of developing these aneurysms.
